# Obituary

**Published:** 1866-12

**Authors:** 


### OBITUARY.

It is with much pain that we have just learned of the death of our intimate friend and professional brother, Dr. A. M. Leslie, of St. Louis, Mo. He died at Memphis, Tenn., on the 30th ult., of cholera. He was attacked suddenly and died in a very short time.

Dr. L. possessed, in a marked degree, those qualities that command the esteem and high regard of all who knew him. He leaves many warm friends in this city, and indeed throughout the country, to mourn his loss. He was ever foremost in doing that which would best subserve the interests of his chosen profession. He was a man of positive character—one who would make his influence felt wherever he was, and in what he undertook. We are advised that a more lengthy sketch of his life will be written than space will here permit.

The bereaved friends and family have our warmest sympathy.

---

DIED—In St. Louis, Mo., on the 29th ult., Dr. John S. Clark, long known to the profession as one of its noblest members. He has, for many years, stood as one of its able supporters and defenders. The Dr., for several years, has been a great sufferer from a neuralgic affection, the influence of which ultimated in his death. His loss will be severely felt. His name has been almost a household word throughout the profession for many years, and he has not died without having done much good.

The profession, with sorrow, will feel tenderly for those who sustained the great loss of husband and father.

